# Adaptation And Use of the Papworth Haemostasis Checklist - Clinical Outcomes Analysis at Hospital Estadual Mário Covas

**DOI:** 10.21470/1678-9741-2022-0305

**Published:** 2023-06-14

**Authors:** Pedro Borghesi Poltronieri, Andrea Cristina Oliveira Freitas, Caroline Hamati Rosa Batista, Jorge Luiz Ribeiro da Luz, Mayara Baschiera Barbosa, Ricardo Siqueira Gonçalves, Maria Carolina Martins Smanio, Adilson Casemiro Pires

**Affiliations:** 1 Faculdade de Medicina, Centro Universitário FMABC, Santo André, São Paulo, Brazil; 2 Centro de Cirurgia Cardiovascular, Hospital Estadual Mário Covas, Santo André, São Paulo, Brazil; 3 Cardiovascular Surgery Department, Faculdade de Medicina, Centro Universitário FMABC, Santo André, São Paulo, Brazil; 4 Cardiothoracic Surgery Department, Faculdade de Medicina, Centro Universitário FMABC, Santo André, São Paulo, Brazil

**Keywords:** Cardiac Surgery, Reoperation, Checklist, Postoperative Hemorrhage, Thoracic Wall

## Abstract

**Introduction:**

Postoperative bleeding is one of the main causes of complications in cardiovascular surgery, which highlights the importance of ensuring adequate intraoperative hemostasis, providing a better patient outcome. This study aimed to improve the prevention of postoperative bleeding in the Cardiovascular Surgery Department of the Hospital Estadual Mário Covas (Santo André, Brazil) using an adapted version of the Papworth Haemostasis Checklist to assess the impact of this standardization on bleeding rate, postoperative complications, reoperation, and mortality.

**Methods:**

This is a non-randomized controlled clinical trial, whose non-probabilistic sample consisted of patients undergoing cardiac surgery in the abovementioned service within a two-year interval. The Papworth Haemostasis Checklist was adapted to the Brazilian laboratory parameters and the questions were translated into Portuguese. This checklist was used before the surgeon started the chest wall closure. Patients were followed up until 30 days after surgery. A P-value < 0.05 was considered statistically relevant.

**Results:**

This study included 200 patients. After the checklist, a reduction in 24-hour drain output, postoperative complications, and reoperation was observed, although statistical significance was not reached. Finally, there was a significant reduction in the number of deaths (8 vs. 2; P=0.05).

**Conclusion:**

The use of the adapted checklist in our hospital proved to be an effective intervention to improve the prevention of postoperative bleeding, with a direct impact in the number of deaths in the study period. The reduction in deaths was possible thanks to the reduction in the bleeding rate, postoperative complications, and reoperations for bleeding.

**Table:** 

Abbreviations, Acronyms & Symbols				
ACT	= Activated clotting time		Hb	= Haemoglobin
AFRVR	= Atrial fibrillation with rapid ventricular response		LA	= Left atrium
AKI	= Acute kidney injury		LIMA	= Left internal mammary artery
AMI	= Acute myocardial infarction		LV	= Left ventricular
BMI	= Body mass index		PA	= Pulmonary artery
CABG	= Coronary artery bypass grafting		RA	= Right atrium
COVID-19	= Coronavirus disease 2019		RSPV	= Right superior pulmonary vein vent
CPB	= Cardiopulmonary bypass		SVG	= Saphenous vein graft
EuroSCORE	= European System for Cardiac Operative Risk Evaluation		TEG	= Thromboelastogram
FBC	= Full blood count			

## INTRODUCTION

Checklists have gained importance in healthcare, being currently widely used in the surgical field to standardize complex processes and reduce the risk of errors. Thus, the use of a checklist in hemostasis procedures is presented as a simple, quick, and easy-to-use tool to prevent complications, yielding a better clinical outcome for patients^[1,2]^.

Postoperative bleeding is one of the main potentially modifiable complications in cardiovascular surgeries^[3]^ since both anemia and the need for transfusions of blood products can significantly increase patients’ mortality and morbidity^[4-7]^. Several studies show that blood transfusion can be harmful by increasing the chance of postoperative infection, myocardial and cerebrovascular ischemia, kidney injury, worse recovery, and death^[8,9]^.

To reduce these modifiable factors, researchers in the United Kingdom developed a multidisciplinary intraoperative checklist, known as the Papworth Haemostasis Checklist. Its assessment is based on two major sections: operative sites and coagulation status. When comparing variables before and after the implementation of this checklist, there was a significant reduction in mediastinal blood loss, rate of return to operating room for hemostasis, and use of blood products. As a secondary outcome, a significant reduction in hospital costs was observed^[1]^. Thus, the benefit of the intervention proposed by the British researchers reinforces the need to standardize criteria related to risk of bleeding in patients undergoing cardiac surgery.

Nowadays, there are not similar checklists aiming to mitigate postoperative bleeding in Brazil, which leads to a mortality rate of 5.6%, that exceeds the global rate of 3%^[10]^. Therefore, the standardization of haemostasis procedures in the form of an easy-to-use tool, as the mentioned checklist, seems to be an adequate way to decrease the bleeding rate of cardiovascular surgery in our country. In this scenario, this study has the objective to improve the prevention of postoperative bleeding in the Cardiovascular Surgery Department of the Hospital Estadual Mário Covas (Santo André, Brazil) with the use of the hemostasis checklist proposed by the Royal Papworth Hospital, in a Brazilian adapted version, to assess the impact of this standardization in bleeding rate (24-hour drain output), postoperative complications, reoperation for bleeding, and mortality.

## METHODS

A non-randomized clinical trial was developed at the Centro de Cirurgia Cardiovascular of the Hospital Estadual Mário Covas in a two-year interval. A non-probabilistic sample was obtained with all patients who underwent cardiac surgery (coronary artery bypass grafting, valve replacement, aortic dissection repair, and ventricular aneurysm repair) within the study period, regardless of sex, age, body mass index (BMI), European System for Cardiac Operative Risk Evaluation (EuroSCORE) II, ejection fraction, heart rate, comorbidities, or surgery priority (elective, urgent, or emergency). Patients who underwent heart transplantation or pulmonary thromboendarterectomy were excluded from the study.

**Fig. 1:**
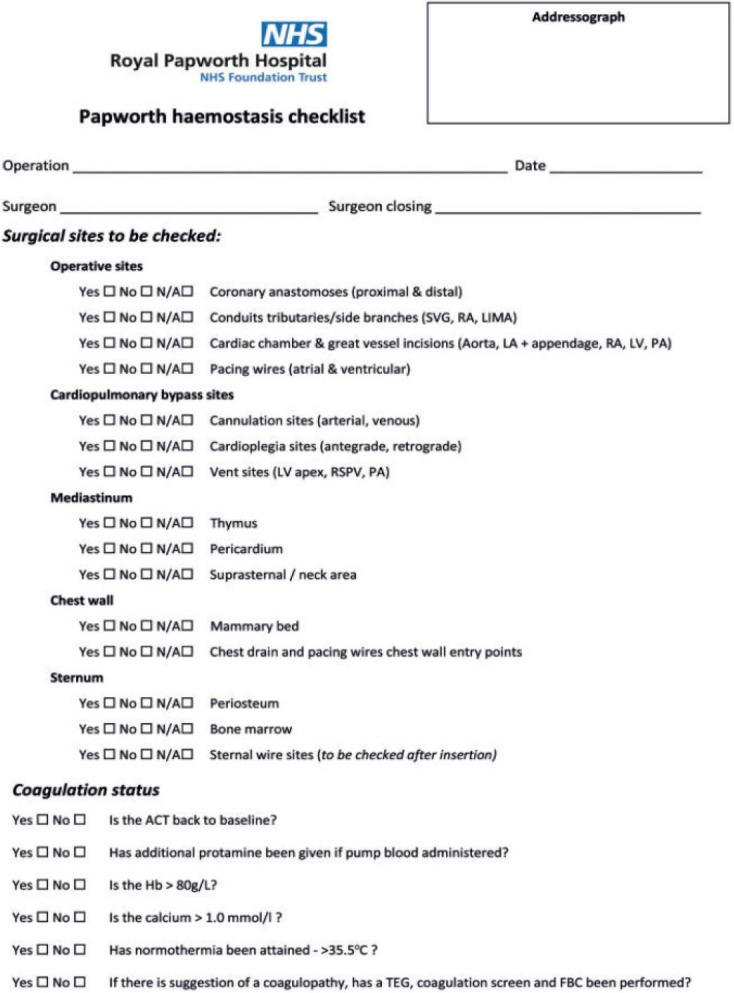
Original checklist by the Royal Papworth Hospital. ACT=activated clotting time; FBC=full blood count; Hb=haemoglobin; LA=left atrium; LIMA=left internal mammary artery; LV=left ventricular; PA=pulmonary artery; RA=right atrium; RSPV=right superior pulmonary vein vent; SVG=saphenous vein graft; TEG=thromboelastogram

Patients were divided into two groups: Group 1 (G1), patients operated without the use of the checklist, and Group 2 (G2), patients in which the hemostasis checklist proposed by the Royal Papworth Hospital was used (Figure 1). G1 patients were enrolled between November 2019 to June 2020. The checklist phase (G2) lasted from November 2020 to June 2021.

**Fig. 2:**
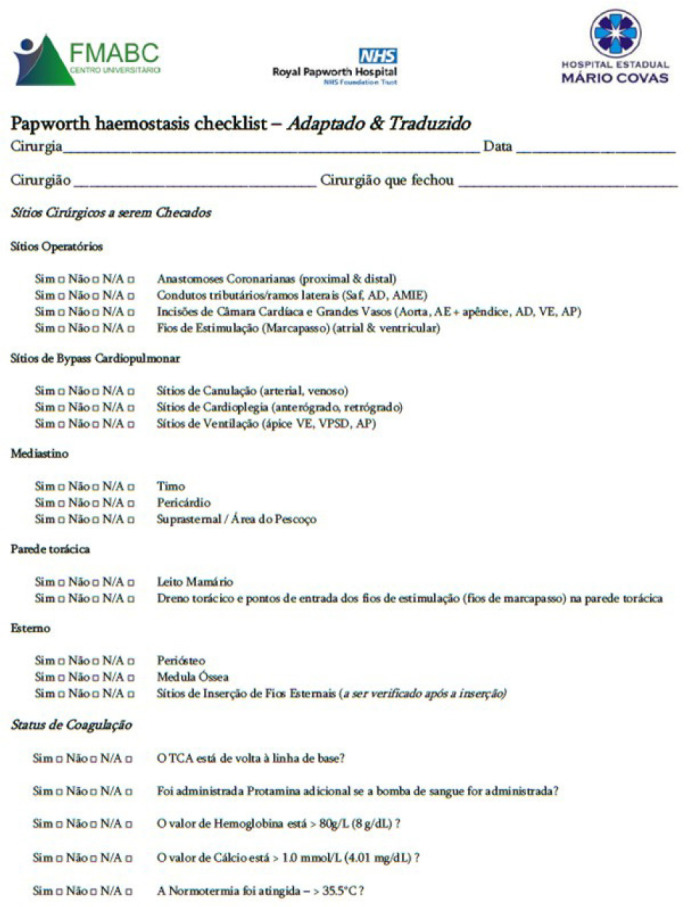
Translated and adapted checklist used in the study. AD=átrio direito; AE=átrio esquerdo; AMIE=artéria mamária interna esquerda; AP=artéria pulmonar; Saf=enxerto de veia safena; TCA=tempo de coagulação ativado; VE=ventricular esquerdo; VPSD=ventilação da veia pulmonar superior direita

The Papworth Haemostasis Checklist was translated into Portuguese and adapted according to the measurement units (g/dL and mg/dL) used in Brazil and at the Hospital Estadual Mário Covas (Figure 2). This adapted version presents the same questions as the original checklist, except for the last question about the use of thromboelastography, which was not available at the hospital. The checklist was used intraoperatively, before the chest wall closure.

The adapted and translated version of the checklist was applied on a two-week period in order to access the feasibility of this new process in our service and to build an awareness culture on the new checklist as a tool to mitigate bleeding complications in our hospital. After this period, the surgical team had a debriefing to optimize the application of this checklist in the intraoperative routine. The major objective of this step was to evaluate how the team would deal with haemostasis revision using a standardized procedure. It was decided to not analyze the patients’ data in this step.

Preoperative data were collected in a standardized way and included demographic information (such as weight, height, and BMI), comorbidities (hypertension, diabetes mellitus, dyslipidemia, and smoking), left ventricular function, and EuroSCORE. In the postoperative evaluation, the chest tube drainage amount (mL) in the first 24 hours, the need for blood transfusions and reoperation for bleeding, intensive care unit and hospital length of stay, postoperative infection, and other complications were observed, as well as postoperative death. Patients were followed up for 30 days after surgery.

All patients were included in the study after written informed consent was obtained. The project was submitted and approved by the Research Ethics Committee of Centro Universitário FMABC (CAAE: 7122920.7.0000.0082).

A descriptive analysis of the data was performed, and, for qualitative variables, the absolute and relative frequencies were calculated. As the variables did not follow a normal distribution by the Shapiro-Wilk test, data were presented as mean and median.

To compare drainage output, amount of blood products, and days of hospitalization between groups, the Mann-Whitney U test was used. In relation to postoperative infection, death, and reoperation, Fisher’s exact test was used. A *P*≤0.05 was considered statistically significant. The analysis was performed using Stata software version 14.0.

## RESULTS

A total of 200 patients were included in the study - 100 patients operated without the use of the checklist (G1) and 100 patients operated with this intervention (G2). Preoperative characteristics of both groups are described in Table 1.

**Table 1:** Clinical characteristics of the patients.

		Group 1	Group 2
		(N=100)	(N=100)
Gender	Female	27	35
	Male	73	65
Age (years)	Mean	62.59	62.23
	Median	63	63
BMI (kg/m^2^)	Mean	27.34	27.19
	Median	27.02	26.47
Surgery priority	Elective	45	46
	Urgent	48	53
	Emergency	7	1
Comorbidities	Hypertension	83	83
	Diabetes	42	46
	Dyslipidemia	19	17
	No comorbidities	16	14
Smoking	Non-smoker	34	25
	History of smoking	66	75
Heart rate	Normal sinus rhythm	91	94
	Atrial fibrillation	4	3
	Other	5	3
Ejection fraction	Preserved	65	63
	Mid-range	22	27
	Reduced	13	10
EuroSCORE II	Low risk (0-2 points)	26	17
	Moderate risk (3-5 points)	48	45
	High risk (6-45 points)	26	38

BMI=body mass index; EuroSCORE=European System for Cardiac Operative Risk Evaluation

Regarding sex distribution in this study, there were a predominance of males and a higher mortality in male patients. Intraoperative characteristics of both groups are described in Table 2.

**Table 2:** Intraoperative characteristics.

		Group 1	Group 2
		(N=100)	(N=100)
Surgery performed	CABG	86	94
	CABG + other surgery	5	0
	CABG + valve replacement	5	2
	Valve replacement	2	2
	Aneurysm/aortic dissection	2	2
Cardiopulmonary bypass (CPB)	CPB use	96	93
	CPB time (mean/median - min)	65.80/33.17	70.58/31.40
	Aortic cross-clamping time (mean/median - min)	52.70/28.93	52.99/25.16

CABG=coronary artery bypass grafting; CPB=Cardiopulmonary bypass

The checklist group (G2) had a shorter hospital stay and a lower bleeding volume, both without statistical significance. However, there was a greater need for blood transfusion. Table 3 shows postoperative state and clinical outcomes.

**Table 3:** Postoperative state.

	Group 1	Group 2	*P*-value
	(N=100)	(N=100)	
Chest tube drainage amount (mL)			0.094
Median (range)	150 (0-1320)	120 (0-2900)	
Mean	214.67	189.5	
Blood products transfusion			0.765
Transfused patients	21	23	
Packed red blood cells (mean)	0.28	0.40	
Platelets (mean)	0.51	0.15	
Cryoprecipitate (mean)	0.33	0.07	
Fresh frozen plasma (mean)	0.22	0.02	
Hospitalization (days)			0.132
Mean (minimum-maximum)	10.64 (1-70)	7.91 (1-40)	
Postoperative infection	19	19	0.571
Complications	62	56	0.388
Reoperation	2	1	0.571
Death	8	2	0.050

Regarding postoperative infection in G1, nine patients had Coronavirus disease 2019 (COVID-19), eight had surgical site infection, eight had pneumonia, and two had urinary tract infection. There were single cases of pulmonary sepsis, sepsis of unknown origin, bloodstream infection, catheter-related infection, pseudomembranous colitis, and *Clostridium difficile*-associated diarrhea. In G2, eight patients had surgical site infection, four had pneumonia, two had urinary tract infection, and two had COVID-19. There were single cases of pulmonary sepsis, bloodstream infection, catheter-related infection, and colitis in G2.

**Fig. 3:**
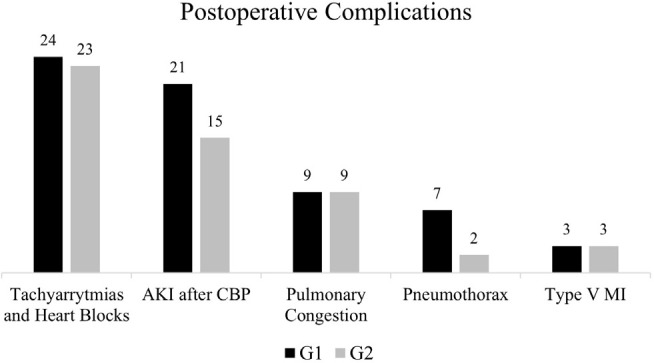
Postoperative complications. Group 1 (G1) includes patients operated without the use of the hemostasis checklist; Group 2 (G2) includes patients operated with the use of the hemostasis checklist. AKI=acute kidney injury; AMI=acute myocardial infarction; CPB=cardiopulmonary bypass

Figure 3 shows the distribution of frequencies of non-infectious complications, of which five stand out.

Within the group of tachyarrhythmias and heart blocks, atrial fibrillation with rapid ventricular response (AFRVR) was the most prevalent, with 15 cases in G1 and 12 in G2.

There was a higher occurrence of reoperation in G1; in one patient, it was due to dehiscence of the aortic cannulation suture for cardiopulmonary bypass (CPB), and in another patient, it was due to cardiac tamponade after bleeding in the right atrium. In G2, the reoperated patient presented bleeding from aorta-saphenous vein anastomosis.

There were eight deaths in G1 and two deaths in G2. Of the eight deaths in G1, two occurred in patients who required reoperation.

## DISCUSSION

There are several studies proposing surgical checklists, considering the benefits of this systematic methodology in reducing complications. However, few studies are focused on cardiac surgery, especially addressing the review of hemostasis processes. Considering this, in our study we chose to use a checklist aimed at this surgical time as proposed by the Royal Papworth Hospital. As in the British study, a reduction in mediastinal bleeding and reoperation for bleeding rates after use of the checklist was observed in the sample of our study, although the transfusion rate did not show a decrease^[1]^.

By standardizing the steps of the final review of hemostasis, an individual failure of any item that goes unnoticed is prevented, which reduced the bleeding volume observed in this sample. However, a lower transfusion rate would be expected in the checklist group, something that was not observed in our study.

Comparing the two groups, the blood products transfusion rate practically remained the same, which may have occurred not only due to the patients’ preoperative state, with a high prevalence of chronic anemia, but also due to absence of a specific protocol such as the one discussed in the study by Bilecen et al^[11]^. These researchers adopted a specific transfusion protocol for cardiovascular surgeries that considered preand post-CPB red blood cell indices, adjusting the level of intervention according to the values found. This protocol reduced the transfusion of packed red blood cells and fresh frozen plasma, with better outcomes. Thus, that it would be interesting to add a hemostasis checklist to a blood transfusion protocol to obtain even more benefits.

Another advantage of the checklist use was the reduction of some complications directly related to a lower postoperative bleeding rate, of which the most significant is acute kidney injury (AKI), the second most common in our study, as shown in Figure 3. Brown et al.^[12]^, in a retrospective study published in 2010, found a direct relationship between increased mortality and development of AKI in the postoperative period of cardiac surgery, and this rate was proportional to the duration of kidney injury. In our study, G2 patients had a lower incidence of AKI and mortality, in agreement with the work of Brown et al^[12]^.

Tachyarrhythmias were frequent complications in our study and followed the same trend found in the literature. Conti et al.^[13]^ showed that supraventricular tachyarrhythmias, especially AFRVR, can occur in 10 to 40% of patients after cardiac surgery, and their incidence is slightly higher in valve repair procedures. As seen in our study, the checklist did not have a significant impact on its reduction, since these conditions are related to the physiologic stress caused by myocardial manipulation during the procedure.

Although not directly related to bleeding rates, a lower incidence of pneumothorax was observed in G2 patients. The application of the checklist during chest wall closure implies a more detailed review of hemostasis and, during this review, a pneumothorax caused by pleural manipulation during the intraoperative period may have been diagnosed and treated early, avoiding the need for future pleural drainage in the intensive care environment, which would lead to longer hospital stay (which occurred with G1 patients).

Some studies showed results similar to ours regarding the reoperation for bleeding rate. Loor et al.^[14]^ observed a significant reduction from 3.1 to 1.9% with the use of a checklist that assessed only sites of bleeding. Regarding reoperation, our service had a 2% rate before the checklist was implemented - within the world range (2-8%) and below the Brazilian average (3.7%). With the checklist, this rate presented values below the world average, reaching 1%^[10,14]^.

Most studies carried out in the last two years have been affected by the COVID-19 pandemic. Surgeries performed during the activity of severe acute respiratory syndrome coronavirus 2 infection resulted in a worsening of surgical complications, especially pulmonary complications, and it was also likely to be associated with hemorrhagic complications. Wang et al.^[15]^ demonstrated an increase in postoperative bleeding, need for blood transfusion, and mediastinal drainage after cardiovascular surgery in patients with COVID-19 when compared to healthy patients. In our study, the institutional protocol postponed surgery in all those who present a COVID-19 positive test preoperatively, so all infected patients acquired the disease in the postoperative period.

In our study, the use of the checklist was accompanied by a statistically significant reduction in deaths. The inclusion of other aspects to be checked regarding the perioperative performance of patients can enhance this result, as described by Spanjersberg et al^[16]^. By proposing a checklist with a broader view and assessment of risk factors, the authors obtained a significant mortality reduction in 120 days.

Stressing the importance of intraoperative bleeding control, in their study, Mazzeffi et al.^[17]^ showed that 19.5% of deaths occurred due to complications from reoperations for bleeding. Corroborating this data and analyzing the impact of transfusion in these patients, Vivacqua et al.^[18,19]^ suggested that transfusion and reoperation provided higher mortality and increased the risk of negative postoperative outcomes.

### Limitations

This study has some limitations. We observed that adherence to the checklist seems to be a challenge. Thus, to achieve all these benefits, team training is essential. Part of the study period took place during the COVID-19 pandemic, which, in addition to delaying data collection and limiting the sample size, made it necessary to exclude deaths of patients diagnosed with COVID-19 in the postoperative period, whose complications could lead to biased results. The absence of thromboelastography in the checklist may have suppressed relevant intraoperative data and possibly affected the blood transfusion rate. The individual decision on indication of blood transfusion in cases of borderline red blood cell indices in this research may have been different from what occurred in the British research that inspired our study. A larger sample, through a multicenter study in Brazil, as well as randomization of the study can improve statistical relevance in the items assessed and refine the analysis of other variables.

## CONCLUSION

The use of Papworth Haemostasis Checklist, adapted and translated into Portuguese, at the Centro de Cirurgia Cardiovascular of Hospital Estadual Mário Covas proved to be a simple and quick intervention to improve the prevention of postoperative bleeding, with an impact on number of deaths in the study period. The reduction in deaths was possible thanks to reduction in bleeding rate, postoperative complications, and reoperations for bleeding. In view of this, our service recommends implementation of similar hemostasis checklists in other centers. A multicenter, randomized study could improve statistical relevance of the items assessed as well as refine the analysis of other relevant variables.

**Table:** 

Authors’ Roles & Responsibilities	
PBP	Substantial contributions to the conception or design of the work; or the acquisition, analysis, or interpretation of data for the work; drafting the work or revising it critically for important intellectual content; final approval of the version to be published
ACOF	Substantial contributions to the conception or design of the work; or the acquisition, analysis, or interpretation of data for the work; drafting the work or revising it critically for important intellectual content; final approval of the version to be published
CHRB	Substantial contributions to the conception or design of the work; or the acquisition, analysis, or interpretation of data for the work; drafting the work or revising it critically for important intellectual content; final approval of the version to be published
JLRL	Substantial contributions to the conception or design of the work; or the acquisition, analysis, or interpretation of data for the work; drafting the work or revising it critically for important intellectual content; final approval of the version to be published
MBB	Substantial contributions to the conception or design of the work; or the acquisition, analysis, or interpretation of data for the work; drafting the work or revising it critically for important intellectual content; final approval of the version to be published
RSG	Substantial contributions to the conception or design of the work; or the acquisition, analysis, or interpretation of data for the work; drafting the work or revising it critically for important intellectual content; final approval of the version to be published
MCMS	Substantial contributions to the conception or design of the work; or the acquisition, analysis, or interpretation of data for the work; drafting the work or revising it critically for important intellectual content; final approval of the version to be published
ACP	Substantial contributions to the conception or design of the work; or the acquisition, analysis, or interpretation of data for the work; drafting the work or revising it critically for important intellectual content; final approval of the version to be published
